# Retraction: Clinical Impact of De-Regulated Notch-1 and Notch-3 in the Development and Progression of HPV-Associated Different Histological Subtypes of Precancerous and Cancerous Lesions of Human Uterine Cervix

**DOI:** 10.1371/journal.pone.0272116

**Published:** 2022-07-21

**Authors:** The PLOS ONE Editors

Following the publication of this article [1], concerns were raised regarding images in Figs 1 and 3. Specifically,

- The Notch-1 panel in Fig 1N of this article [1] appears similar to the PML panel in Fig 1F of [2], flipped horizontally and with color levels adjusted.
- Images described as the original raw data were provided for Fig 3. These images appear to present the same data as the published panels without adjustment to contrast or brightness. The following issues were identified:
  ○ In the images provided as raw data for Fig 3B, 3C and 3D, there appear to be vertical discontinuities suggestive of splice lines.
  ○ In the image for Fig 3B, lanes C2 and N appear similar to each other despite representing different conditions.
  ○ In the image for Fig 3D, lanes N1, N2 and N appear similar to each other despite representing different samples.

These issues were discussed with the corresponding author and first author.

The first author stated that the Notch-1 panel in Fig 1N of this article [1] was included in error. They provided a replacement image for Notch-1 and raw image data for the replacement panel. Raw image data were no longer available for Notch-3 and β-actin panels in Fig 1N, and cropped images were provided.

The first and corresponding authors acknowledged the concerns in the images described as raw data for Fig 3. They clarified that these files were not the raw data and were submitted in error. Additional image data underlying Fig 3 were provided which clarified that some of the observed splice lines were caused by the re-ordering of lanes.

The concerns regarding similarities between lanes within Fig 3B and 3D remain unresolved. The authors offered to repeat the experiment; however, while these concerns remain unresolved, the journal does not consider alternative or repeat experimental data to be sufficient to resolve the issues.

Given the concerns regarding the integrity of Fig 3, the *PLOS ONE* Editors retract this article.

RT and MB agree with the retraction and apologize for the issues with the published article. All other authors either did not respond directly or could not be reached.
